# Efficacy and safety of Xiao-ai-ping injection add-on therapy to chemotherapy in patients with non-small cell lung cancer: A systematic review and meta-analysis

**DOI:** 10.1097/MD.0000000000035483

**Published:** 2023-10-06

**Authors:** Andong Li, Shilin Liu, Hui Zhang, Minghao Lin, Lijiao Guo, Chengbo Yuan, Zhenyu Li, Jianan Xu, Tan Wang

**Affiliations:** a Changchun University of Chinese Medicine, Changchun, Jilin Province, China; b Pulmonology, First Affiliated Hospital to Changchun University of Chinese Medicine, Changchun, Jilin Province, China.

**Keywords:** Xiao-ai-ping injection, chemotherapy, non-small cell lung cancer, randomized control trial, meta-analysis

## Abstract

**Background:**

Xiao-ai-ping injection (XAPI) combined with chemotherapy has potential efficacy and less side effects in the treatment of non-small cell lung cancer (NSCLC). At present, there are many clinical studies on XAPI combined with chemotherapy in the treatment of NSCLC, but the results are different. The purpose of this study was to evaluate the efficacy and safety of XAPI combined with chemotherapy in the treatment of NSCLC by meta-analysis system.

**Methods:**

The databases to be searched include PubMed, Cochrane Library, Web of Science, Chinese National Knowledge Infrastructure, Chinese Biomedical Literature Database, Wanfang database, Chinese Scientific Journal Database, and so on. In addition, relevant journals and magazines will manually search in various fields as supplements. The search date is set from the establishment of the database until July 8, 2023. The 2 researchers will use Endnote X9 software for literature screening and data extraction and independently evaluate the quality. We then assessed the quality and risk of inclusion in the study and observed outcome indicators.

**Results:**

A total of 28 trials were included in this study, 1947 patients with NSCLC (974 receiving XAPI combined chemotherapy and 973 receiving chemotherapy alone). The results of meta-analysis showed that: Objective tumor response rate of NSCLC (*P* < .00001). Improvement in Karnofsky performance score of NSCLC (*P* < .00001). Quality of life score of NSCLC (*P* < .00001). The result of CD3 + (*P* < .00001). The result of CD4 + (*P* < .00001). The result of CD8 + (*P* < .00001). The result of CD4+/CD8 + (*P* = .0001). Leukopenia (*P* < .00001). Thrombocytopenia (*P* < .00001). Hemoglobin decrease (*P* < .00001). Liver function (*P* = .04). Nausea and vomiting (*P* < .00001).

**Conclusion:**

Our meta-analyses demonstrated that XAPI adjunct with chemotherapy can improve the patient quality of life, reduce adverse reactions, and enhanced immune function, the treatment is effective and high safety. Which suggests that it might be used for NSCLC. However, a large sample of randomized controlled trials are needed to further study the long-term efficacy of XAPI.

## 1. Introduction

Malignant tumor is one of the main causes of death in the world,^[1]^ among which lung cancer is the malignant tumor with the highest morbidity and mortality in the world.^[2]^ It has become a burden on human health.^[3]^ According to research statistics, about 2.2 million new lung cancer cases and 1.6 million lung cancer patients died in 2020,^[4]^ and non-small cell lung cancer (NSCLC) accounted for about 85% of all lung cancer.^[5]^ Although some progress has been made in the treatment of NSCLC in the past 20 years, it is still an international problem to be solved urgently.^[6]^ At present, chemotherapy is the main method for the treatment of advanced NSCLC. Due to the low early diagnosis rate of NSCLC, about 75% of patients with NSCLC were found in the middle and advanced stage, missed the best period of surgery, and the 5-year survival rate was 2%.^[7]^ Chemotherapy is the best means to maintain the treatment of patients with advanced NSCLC, but its adverse reactions often occur, which bring pain to patients in the course of disease, and it is difficult to get through the prescribed treatment cycle.^[8]^ Therefore, it is necessary to formulate new strategies to maximize the control of tumors in patients with NSCLC and minimize adverse reactions.

In China, because of its multi-target characteristics, the active components of traditional Chinese medicine can play a role in tumor treatment in many ways. Many patented traditional Chinese medicines have great potential efficacy as adjuvant drugs. It plays a role in reducing toxicity and increasing efficiency in the treatment of tumor, so it is very popular in clinical application. Xiao-ai-ping injection (XAPI) comes from the preparation of traditional Chinese medicine Tongguanteng, and its main component is the extract of Tongguanteng.^[9]^ Tongguanteng is a folk herbal medicine in southern China, which has been used in the treatment of cancer gradually since 1969 because it is effective in the treatment of malignant tumors. Later, after a series of pharmacological and clinical studies, Xiaoaiping was approved to be put on the market in 1984 and became a national officially listed drug. It has been widely used in clinic for many years, often used in palliative treatment, and combined with radiotherapy and chemotherapy to improve the efficacy of lung cancer, gastric cancer, liver cancer, leukemia and other malignant tumors. The main active components of Tongguanteng,^[9]^ including C21 steroidal saponins, alkaloids, terpenoids, polysaccharides, alcohols and other chemical constituents, and it not only has the effect of anti-tumor and inhibition of invasion,^[10]^ but also has the functions of regulating immune function, anti-tumor, protecting liver and diuresis and reducing blood pressure.^[11]^ In recent years, there are many clinical studies on Xiaoaiping injection combined with chemotherapy, but there is a lack of large-scale clinical studies, and there are differences between the results of studies. Therefore, this study uses the method of meta analysis to comprehensively evaluate the status of XAPI as an adjuvant treatment of NSCLC in the treatment of advanced NSCLC, and systematically evaluate its clinical efficacy and safety, so as to provide reference for clinical decision-making.

## 2. Materials and methods

### 2.1. Objectives and registration

This review aimed to evaluate the Efficacy and safety of XAPI add-on therapy to chemotherapy in patients with NSCLC. Our goal is to publish this review in a peer-reviewed journal. This article will follow the preferred reporting items for systematic reviews and meta analysis statement.^[12]^ The protocol for this review and meta-analysis has been registered on the International Prospective Register of Systematic Reviews with the registration number CRD42023443648.

### 2.2. Data sources and retrieval strategy

These studies are conducted using PubMed, Cochrane Library, Web of Science, Chinese National Knowledge Infrastructure, Chinese Biomedical Literature Database, Wanfang database, Chinese Scientific Journal Database, and so on. The time for inclusion of the literature is for each database to be established until July 8, 2023. Before literature retrieval, we conducted professional training and study on literature retrieval skills and matters needing attention, and after 2 pre-checks, finally formulated the retrieval strategy. We retrieved the database through a combination of subject words and free words, using PubMed as an example, the specific retrieval strategy is shown in Table 1, and the retrieval formula is appropriately adjusted according to the characteristics of other databases.

**Table 1:** Search strategy for Pubmed.

Number	Search terms
#1	“Carcinoma, Non-Small-Cell Lung”[Mesh]
#2	(Carcinoma, Non Small Cell Lung[Title/Abstract]) OR (Carcinomas, Non-Small-Cell Lung[Title/Abstract]) OR (Lung Carcinoma, Non-Small-Cell[Title/Abstract]) OR (Lung Carcinomas, Non-Small-Cell[Title/Abstract]) OR (Non-Small-Cell Lung Carcinomas[Title/Abstract]) OR (Non-Small-Cell Lung Carcinoma[Title/Abstract]) OR (Non-Small Cell Lung Carcinoma[Title/Abstract]) OR (Carcinoma, Non-Small Cell Lung[Title/Abstract]) OR (Non-Small Cell Lung Carcinoma[Title/Abstract]) OR (Non-Small Cell Lung Cancer[Title/Abstract]) OR (Nonsmall Cell Lung Cancer[Title/Abstract])
#3	#1 OR #2
#4	“Marsdeniae tenacissimae”[Mesh]
#5	(Marsdenia tenacissima extract[Title/Abstract]) OR (Xiao-ai-ping injection[Title/Abstract]) OR (Xiaoaiping[Title/Abstract]) OR (Xiao-ai-ping[Title/Abstract]) OR (Tongguanteng[Title/Abstract]) OR (Tongguanteng extract[Title/Abstract]) OR (Tongguangteng[Title/Abstract]) OR (Tongguangteng extract[Title/Abstract]) OR (XAP[Title/Abstract]) OR XAP (injection[Title/Abstract]) OR (XAPI[Title/Abstract]) OR (MTE[Title/Abstract])
#6	#4 OR #5
#10	“randomized controlled trial”[Publication Type] OR “controlled clinical trial”[Publication Type]
#11	“Randomized”[Title/Abstract] OR “randomly”[Title/Abstract]
#12	#10 OR #11
#13	#3 AND #6 AND #12

We will also search the World Health Organization International Clinical Trial Registration platform to track ongoing or completed clinical studies. If the included studies lacked relevant data, we will contact the relevant researchers to obtain the required data. If the required information is not available, the data will be excluded from the analysis and are explained in the discussion section. Simultaneously, we manually searched the list of all references from related system reviews to avoid missing high-quality randomized controlled trials (RCT) that met the criteria.

### 2.3. Eligibility criteria

We will determine the inclusion and exclusion criteria for this study in accordance with the population, intervention, comparison, outcome, study design principles.

#### 2.3.1. Types of studies.

All included literatures were clinical RCTs of XAPI add-on therapy to chemotherapy in patients with NSCLC. The language was restricted to Chinese and English. We will exclude other studies, such as observational studies, non-randomized controlled studies, animal trials, crossover studies, personal experience summaries and case reports.

#### 2.3.2. Types of participants.

Including of any gender, any profession or ethnicity, and any age ≥ 18 patients with NSCLC.

#### 2.3.3. Types of interventions and comparisons.

The treatment group received XAPI add-on therapy to chemotherapy. The control group was treated with chemotherapy alone. There are no requirements for the time, frequency, dose, and course of treatment.

### 2.4. Types of outcome measures

The primary outcomes were as follows: Objective tumor response rate (ORR): ORR = (complete response + PR)/total number of cases × 100%. According to the objective efficacy criteria of WHO antineoplastic drugs, the efficacy was divided into complete response (complete response, It means that the lesion disappeared completely for more than a month), partial response (PR, The mass has shrunk by more than 50% for no <4 weeks.), stable disease (The mass shrinks by <50% or enlarges by <25%.) and progressive disease (PD, One or more lesions increase by more than 25% or new lesions appear); Karnofsky performance score (KPS); Quality of life score; Immune function (the percentage of CD3+, CD4+, CD8+, CD4+/CD8+); Adverse effects, including changes in white blood cells, platelets, hemoglobin, liver function and improvement in nausea and vomiting symptoms. If other results are reported in the eligible studies, they will be extracted and reported.

### 2.5. Data collection and analysis

#### 2.5.1. Selection of studies.

Two researchers (Andong Li and Shilin Liu) conducted literature retrieval according to the retrieval strategy developed in advance, and screened the literature according to the inclusion and exclusion criteria. The literature retrieved from the database is imported into the Endnote X9 software (Derived from the Thomson Corporation Corp, Stanford, CT) and a preliminary screening is performed by reading the title and abstract. Delete all meeting minutes, guides, letters and other documents, as well as duplicate documents. This is followed by downloading and reading of the full text for further screening. The 2 researchers cross-check that any differences can be resolved through consultation, and if there is no consensus, consult with a third researcher (Tan Wang) and make a final agreement.

#### 2.5.2. Data extraction.

Two researchers extracted independent data according to the predesigned extraction table of the Cochrane Handbook for Systematic Reviews of Interventions. The extracted data include 4 aspects: basic information included in the literature, basic characteristics of patients, intervention measures, and outcome indicators. If there is missing or unclear information in an RCT, we will attempt to contact the author of the literature to obtain data. Any dissecting opinions will be dealt with through consultation or consultation with a third researcher to resolve the differences.

### 2.6. Risk of bias assessment

The 2 researchers will use the Cochrane risk bias assessment tool to assess all bias risks included in the study and cross-validate the results. If there is a difference of opinion, decide in consultation with the third researcher until a consensus is reached. The specific evaluation includes the following 6 aspects: random sequence generation method; whether allocation concealment is used; whether the subject and the intervention provider are blinded; whether the result evaluator is blind; whether the result data are complete; whether selective results are reported and other sources of bias. Each aspect will be rated as uncertain, low or high-risk.

### 2.7. Statistical analysis

We will use the ReviewManager v5.4 software (The Nordic COCHRANE Centre, Copenhagen, Denmark. https://training.cochrane.org/online-learning/core-software/revman/rmw-development) for meta-analysis. *P* < .05 is considered to be statistically significant. Two researchers will extract and input the data independently, which will be examined by a third researcher, and the other 2 researchers will conduct the data accounting. Statistical analysis of dichotomous variables are represented by the weighted mean difference (MD) and 95% confidence interval (95%CI). Statistical analysis of continuous variable is expressed by relative risk (RR) and 95%CI.

### 2.8. Assessment of heterogeneity

We will use chi-square statistics and I^2^ to evaluate the statistical heterogeneity between studies. If I^2^ ≦ 50% or *P* > .10, indicating that the heterogeneity test is not significant, a fixed-effect model is used. If I^2^ > 50%, or *P* < .10, there is statistical heterogeneity, and the random effects model is used to analyze the data. In addition, owing to differences in heterogeneity, we will conduct subgroup or sensitivity analyses to identify potential causes.

### 2.9. Subgroup analysis

Subgroup analysis is used to explore potential factors leading to significant heterogeneity. If a high degree of heterogeneity is found in the study, we conduct a subgroup analysis to explore the source of heterogeneity. To explore the differences in race, age, country, gender, different forms of intervention, and so on.

### 2.10. Sensitivity analysis

Sensitivity analysis will be used to test the stability and robustness of the findings. Studies with a high risk of bias or numerical distance from the remaining data are excluded. If heterogeneity remains unchanged after excluding individual literatures, it shows that our research results are relatively robust. If heterogeneity changes after excluding a study, it may be a source of heterogeneity. We will further analyze and explain the root causes of heterogeneity.

### 2.11. Publication bias

If more than 10 articles are included, the risk of publication bias is analyzed using a funnel diagram. When the funnel diagram is obviously asymmetric, it indicated publication bias.

### 2.12. Ethics and dissemination

Patients and the public were not required to participate in the research design, data statistical process and results of this study. Informed consent and ethical approval were not required. In addition, the results of this study will be disseminated through peer-reviewed publications.

## 3. Results

### 3.1. Data collection

**Figure 1.**
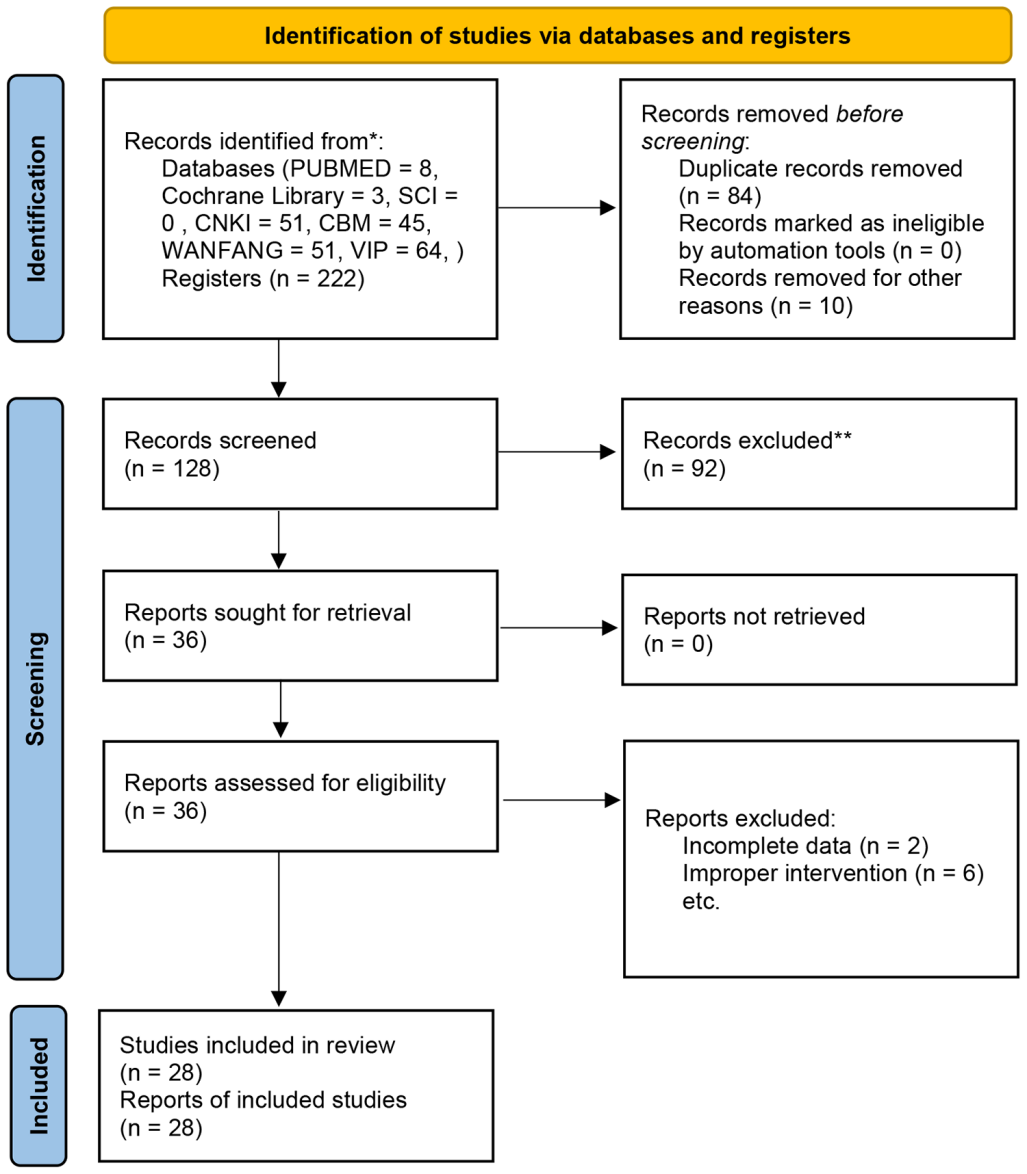
Guidelines flow diagram.

We searched a total of 222 possible relevant articles from 7 databases. 84 repetitive literatures were deleted after preliminary screening by Endnote X9 software. 84 repetitive literatures were deleted after preliminary screening by EndnoteX9 software. After reading the topics and abstracts one by one, 110 articles were excluded because of improper intervention, animal or in vitro experiments and reviews. After further reading, the full text was screened according to the designated inclusion criteria, and finally included 28 articles. A flow diagram of the selection process is presented in Figure 1.

### 3.2. Description of included studies

Included 28 studies published between 2009 and 2020.^[13–40]^ All these studies were carried out in China. Overall, 1947 patients with NSCLC (974 receiving XAPI combined chemotherapy and 973 receiving chemotherapy alone) were identified and included in this meta-analysis. XAPI is given intravenously. The dose of XAPI was 4~80 mL/day, the course of treatment was 1 to 4 cycles. In these studies, the most common schemes are gemcitabine + cisplatin,^[18–22,29,31–37,39]^ taxol + cisplatin^[26–28,30,36,40]^ and navelbine + cisplatin^[23,33–36]^ schemes. According to the pathological TNM staging of NSCLC, all the patients included in the study were III to IV stage diseases. The demographic and clinical baseline characteristics of the study, such as the name of the first author, year of publication, interventions and results, are shown in Table 2.

**Table 2:** Description of included studies.

Study ID	Sample size		Mean age/age		Intervention		Outcomes
	T	C	T	C	T	C	
Qu Hong ying 2015^[13]^	30	30	55.4	55.4	Docetaxel + XAPI	Docetaxel	①②⑧⑪⑫
Wang Shuaibing 2013^[14]^	25	25	58.5	58.5	Docetaxel + XAPI	Docetaxel	①②⑧⑨⑪⑫
Wu Honglin 2016^[15]^	50	50	43.5 ± 6.9	43.5 ± 6.9	Docetaxel + XAPI	Docetaxel	①②⑫
Zhang Zhihua 2012^[16]^	16	16	52.5 ± 1.2	52.5 ± 1.2	Pemetrexed + XAPI	Pemetrexed	①⑫
Hu Yanhui 2019^[17]^	39	39	67.87 ± 7.12	68.37 ± 6.97	TC + XAPI	TC	①③④⑤⑥⑦
Wang Yantao 2012^[18]^	28	28	56	56	GP + XAPI	GP	①②⑧⑨⑫
Zhang Fengyun 2011^[19]^	24	24	65	65	GP + XAPI	GP	①②④⑤⑥⑦⑧⑨⑩
Liu Jinrong 2016^[20]^	30	30	59	57	GP + XAPI	GP	①②③⑧⑨⑩⑫
Li Qinglin 2016^[21]^	36	36	57	56	GP + XAPI	GP	①②⑨⑩⑪⑫
Zhang Xinglong 2019^[22]^	40	40	56.5 ± 2.3	55.5 ± 2.3	GP + XAPI	GP	①③
Wang Ken 2009^[23]^	28	28			NP + XAPI	NP	①②④⑤⑥⑦⑧⑨⑩
Gao Zhihao 2016^[24]^	34	34	71.4 ± 3.6	65.7 ± 4.3	TC + XAPI	TC	①②⑨⑫
Li Yali 2015^[25]^	33	32	59.5	61.2	TC + XAPI	TC	①②③⑧⑨⑩⑫
Mei Chaorong 2015^[26]^	30	33	60.27 ± 11.44	60.73 ± 10.85	TP + XAPI	TP	①②⑧⑨⑩⑪⑫
Xia Guoan 2013^[27]^	39	40	39–77岁	40–76岁	TP + XAPI	TP	①②
Shen Liwei 2015^[28]^	28	28	56	56	TP + XAPI	TP	①②⑧⑨⑩
Liu Shuzhen 2011^[29]^	36	35	48	48	GP + XAPI	GP	⑤
Yao Jun 2016^[30]^	53	52	56.8 ± 3.1	57.4 ± 2.6	TP + XAPI	TP	①③⑧⑨⑩⑪⑫
Liu Aifang 2016^[31]^	36	36	56.43 ± 6.54	57.86 ± 6.42	GP + XAPI	GP	①②
Fang Huan 2013^[32]^	43	43	-	-	GP + XAPI	GP	①⑧⑨⑩⑫
Zhang Ruixing 2012^[33]^	35	33	63.5	63.5	GP/NP + XAPI	GP/NP	①②⑧⑨⑩⑪⑫
Song Yu 2016^[34]^	40	40	65.3 ± 2.8	65.9 ± 2.6	GP/NP + XAPI	GP/NP	①⑧⑨⑩⑫
Xu Jianjian 2017^[35]^	42	42	59.63 ± 6.05	58.31 ± 6.49	GP/NP + XAPI	GP/NP	①②
Li Xiangjing 2020^[36]^	43	43	61	63	GP/NP/TP + XAPI	GP/NP/TP	①④⑦
Hu Xiaolin 2017^[37]^	53	53	60.2 ± 11.4	61.4 ± 11.5	GP + XAPI	GP	①⑧⑨⑩⑪⑫
Yang Wanquan 2013^[38]^	37	37	64	64	Docetaxel + Oxaliplatin + XAPI	Docetaxel + Oxaliplatin	①②⑧⑨⑩⑪⑫
Liu Zan 2016^[39]^	44	44	54.26 ± 3.98	55.19 ± 4.10	GP + XAPI	GP	①⑤⑥⑦⑧⑨⑪⑫
Rao Shijun 2018^[40]^	40	40	63	63	TP + XAPI	TP	①

① ORR (objective tumor response rate); ② KPS (Karnofsky performance scor); ③ QOL (quality of life score); ④ CD3+; ⑤ CD4+; ⑥ CD8+; ⑦ CD4+/CD8+; ⑧ leukopenia; ⑨ thrombocytopenia; ⑩ hemoglobin decrease; ⑪ liver function; ⑫ nausea and vomiting.

GP = gemcitabine + cisplatin, NP = navelbine + cisplatin, TP = taxol + cisplatin; outcomes, TC = taxol + carboplatin, XAPI = Xiao-ai-ping injection.

### 3.3. Quality assessment of studies

**Figure 2.**
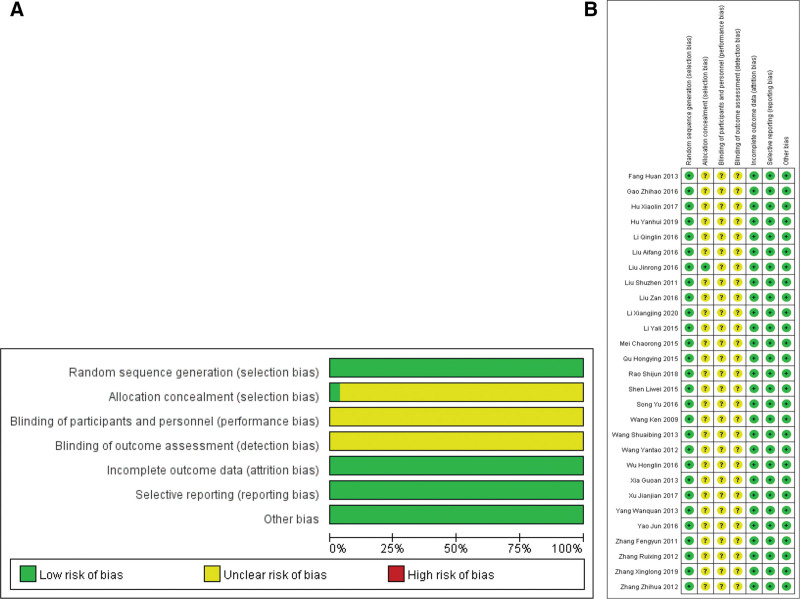
Risk of bias graph (A) and risk of bias summary (B).

We used the Cochrane risk bias assessment tool to assess the risk of bias in each study. All 28 studies described randomization, but only 6 studies^[16,17,21,26,32,38]^ described the specific process of randomization. Blind method or distributive concealment is not mentioned in all studies. Selective reporting bias was not observed, and the existence of other biases was not clear. The quality evaluation and risk bias assessment included in the study are shown in Figure 2.

### 3.4. Outcome indicators

#### 3.4.1. Objective tumor response rate (ORR).

**Figure 3.**
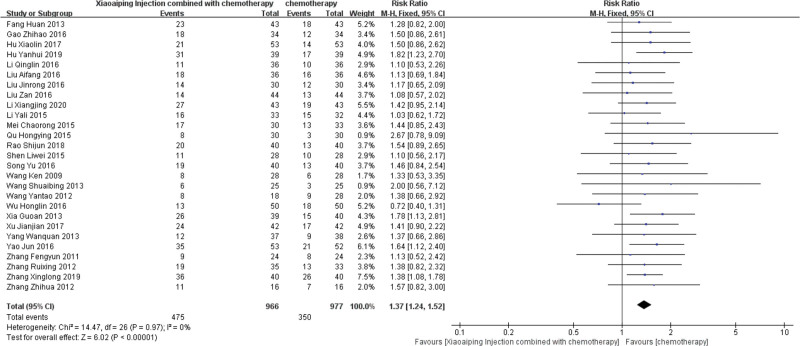
Forest plot of Objective tumor response rate.

In the included studies, 27 trials^[13–28,30–40]^ reported the ORR of the 2 groups, a total of 1947 patients, including the treatment group (n = 973) and the control group (n = 974). There was no statistical difference between the studies (*P* = .97 and I^2^ = 0.0%), so the fixed effect model was used. The results of meta analysis showed that the diamond-shaped square representing ORR was located on the right side of the equivalent vertical line. Compared with the control group, the combination of XAPI with chemotherapy was significantly better than the control group with chemotherapy alone, and the difference was statistically significant (RR = 1.37, 95%CI (1.24, 1.52), *P* < .00001) (Fig. 3).

#### 3.4.2. Improvement in KPS.

**Figure 4.**
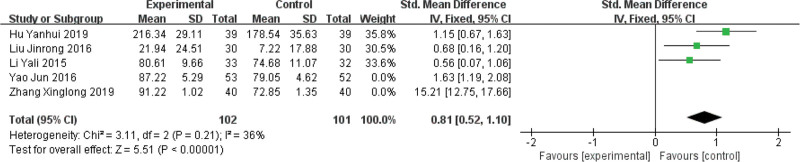
Forest plot of improvement in Karnofsky performance score (KPS).

Heterogeneity test: a total of 5 articles^[17,20,22,25,30]^ were included, and the KPS were analyzed by meta. Heterogeneity test, I^2^ = 97% > 50%, and *P* < .01 for the *Q* test. This shows that the heterogeneity between the selected literature for this study is statistically significant and needs to be searched for heterogeneity. Through the sensitivity analysis of 5 articles, it is found that Zhang Xinglong 2019^[22]^ and Yao Jun 2016^[30]^ have great influence on heterogeneity. The 2 articles were deleted and heterogeneity was tested again. Meta analysis was conducted using fixed effect model, and the results were statistically homogeneous (χ^2^ = 3.11, *P* = .21, I^2^ = 36%). Fixed effect model was used to analyze MD = 0.81, 95%CI (0.52, 1.10), *P* < .00001. It is suggested that the curative effect of XAPI combined with chemotherapy is significantly better than that of the control group (Fig. 4).

#### 3.4.3. Quality of life score.

**Figure 5.**
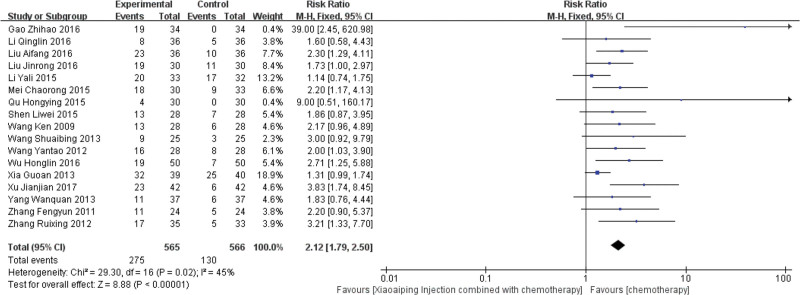
Forest plot of Quality of life score.

A total of 17 trials^[13–15,18–21,23–28,31,33,35,38]^ were included. The results of meta analysis showed that the homogeneity test (χ^2^ = 29.30, *P* = .02, I^2^ = 45%), was analyzed by random effect model (RR = 2.12, 95%CI [1.79, 2.50], *P* < .00001), suggesting that XAPI combined with chemotherapy was significantly better than the control group in improving the quality of life (Fig. 5).

#### 3.4.4. Immune function.

**Figure 6.**
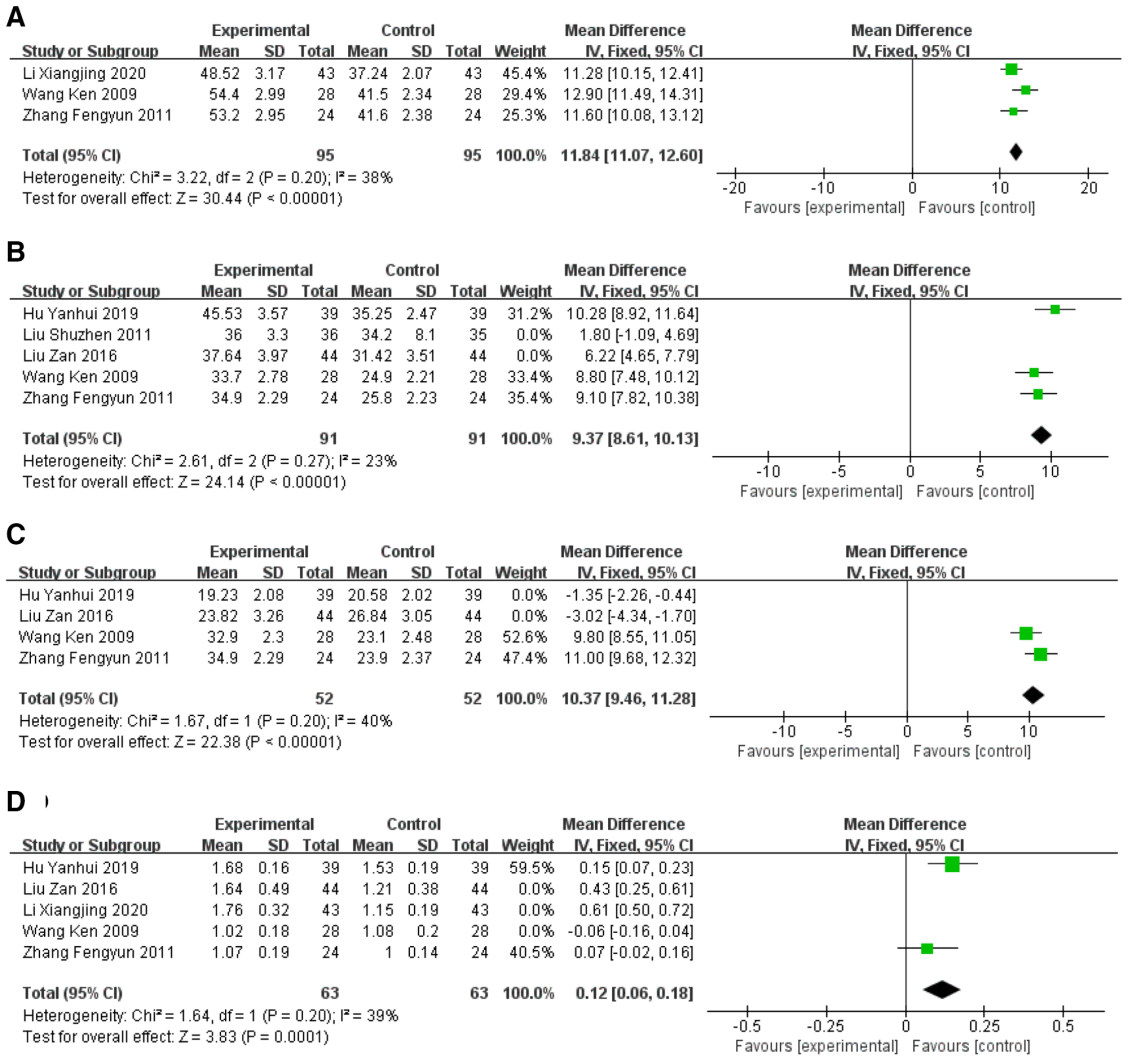
Forest plots of Immune Function (A: the result of CD3+; B: the result of CD4+; C: the result of CD8+; D: the result of CD4+/CD8+).

A total of 6 articles^[17,19,23,29,36,39]^ reported immune function, among which the immune indexes involved were CD3+, CD4+, CD8 + and CD4+/CD8+. Three of them^[19,23,36]^ reported CD3+. After meta analysis, the results were statistically homogeneous (χ^2^ = 3.22, *P* = .20, I^2^ = 38%), analyzed by fixed effect model, MD = 11.84, 95% CI (11.07, 12.60), *P* < .00001. It is suggested that the result of CD3 + in the experimental group is better than that in the control group after treatment (Fig. 6A).

Five articles^[17,19,23,29,39]^ were included in the reports on CD4+, and the CD4 + values were analyzed. The results were statistically homogeneous (χ^2^ = 2.61, *P* = .27, I^2^ = 23%). The fixed effect model was used to analyze, MD = 9.37, 95% CI (8.61, 10.13), *P* < .00001. It is suggested that the result of CD4 + in the experimental group is higher than that in the control group after treatment (Fig. 6B).

Four articles^[17,19,23,39]^ reported on CD8 + were included, and the results of meta analysis showed that the heterogeneity test (χ^2^ = 422.32, *P* < .00001), it showed that the included literature had obvious heterogeneity. By observing the distribution of CIs among the studies, it was found that the data of CD8 + studied by Hu Yanhui 2019^[17]^ and Liu Zan 2016^[39]^ were quite different from other studies. After excluding the 2 studies, the heterogeneity test (χ^2^ = 1.67, *P* = .20, I^2^ = 40%) was carried out again, and the studies were statistically homogeneous. The fixed effect model was used to analyze the results. The results showed that MD = 10.37, 95%CI (9.46, 11.28), *P* < .00001. It is suggested that the result of CD8 + in the experimental group is higher than that in the control group after treatment (Fig. 6C).

Five articles^[17,19,23,36,39]^ reported on CD4+/CD8 + were included, and the results of meta analysis showed that the heterogeneity test (χ^2^ = 92.61, *P* < .00001) showed that the included literature had obvious heterogeneity. By observing the distribution of CI among various studies, it was found that the CD4+/CD8 + data of Hu Yanhui 2019,^[17]^ Liu Zan 2016^[39]^ and Li Xiangjing 2020^[36]^ were quite different from other studies. After excluding the 3 studies, the heterogeneity test (χ^2^ = 1.67, *P* = .20, I^2^ = 49%) was carried out again, and each study was statistically homogeneous. The fixed effect model was used for statistical analysis. The results showed that MD = 0.12, 95%CI (0.06, 0.18), *P* = .0001. It is suggested that the result of CD4+/CD8 + in the experimental group is higher than that in the control group after treatment (Fig. 6D).

#### 3.4.5. Adverse effects.

**Figure 7.**
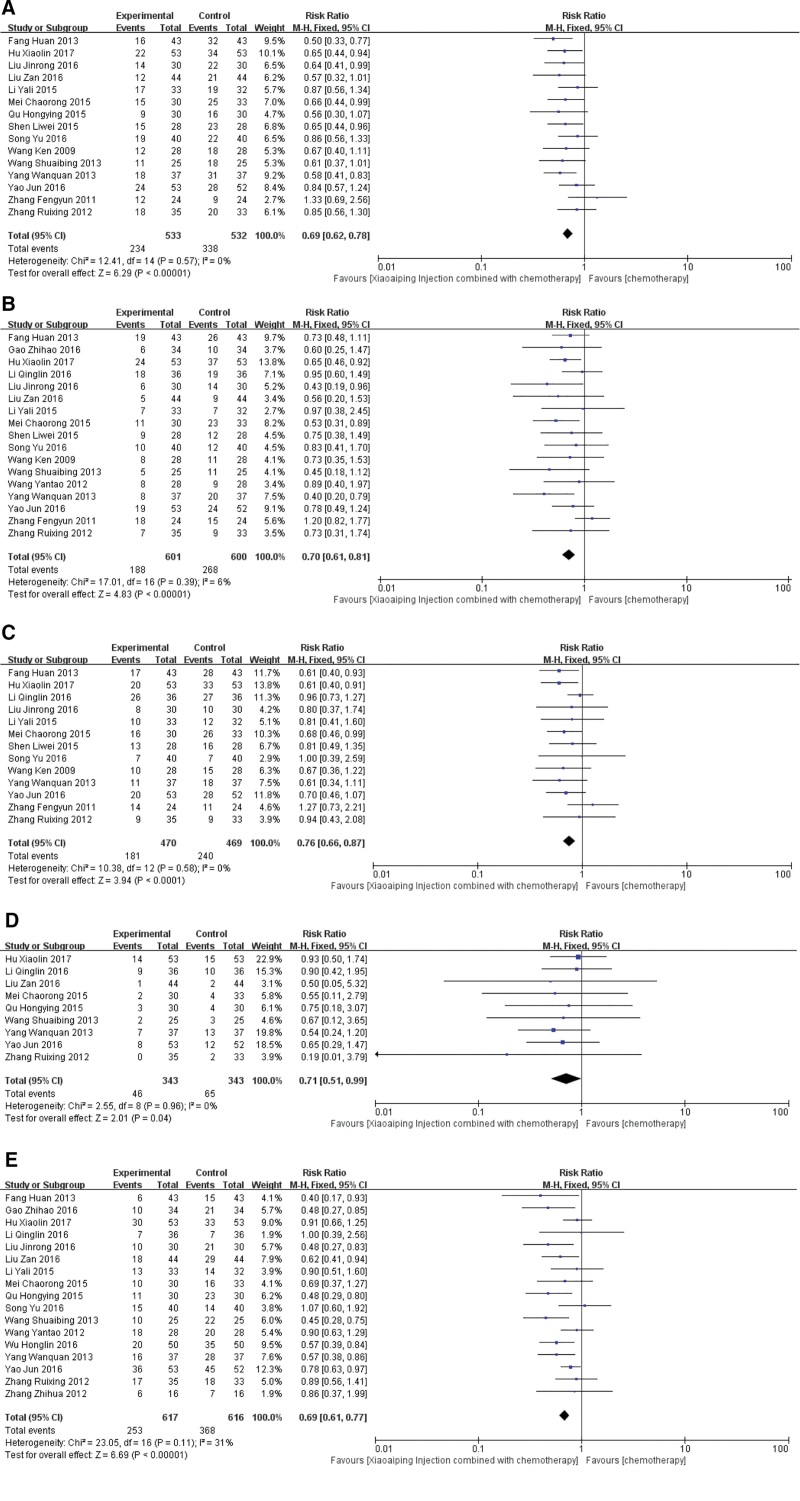
Forest plots of Adverse Effects (A: leukopenia; B: thrombocytopenia; C: hemoglobin decrease; D: liver function; E: nausea and vomiting).

The adverse reactions mentioned in the final included articles mainly included leukopenia, thrombocytopenia, hemoglobin decrease, liver function and digestive system reactions. Among them, 15 articles^[13,14,19,20,23,25,26,28,30,32–34,37–39]^ about leukopenia were included. Homogeneity test (χ^2^ = 12.41, *P* = .57, I^2^ = 0%), using fixed effect model analysis, RR = 0.69, 95%CI (0.62, 0.78), *P* < .00001. It is suggested that the number of leukopenia in the treatment group is lower than that in the control group after treatment (Fig. 7A).

Seventeen articles^[14,18–21,23–26,28,30,32–34,37–39]^ reported thrombocytopenia, homogeneity test (χ^2^ = 17.01, *P* = .39, I^2^ = 6%), using fixed effect model analysis, RR = 0.70, 95%CI (0.61, 0.78), *P* < .00001. It is suggested that the number of thrombocytopenia in the treatment group is significantly lower than that in the control group after treatment (Fig. 7B).

Thirteen articles^[19–21,23,25,26,28,30,32–34,37,38]^ reported hemoglobin decrease, homogeneity test (χ^2^ = 10.38, *P* = 0581, I^2^ = 0%), using fixed effect model analysis, RR = 0.76, 95%CI (0.66, 0.87), *P* < .00001. It is suggested that the decrease of hemoglobin in the treatment group is lower than that in the control group after treatment (Fig. 7C).

A total of 9 articles^[13,14,21,26,30,33,37–39]^ reported abnormal liver function, homogeneity test (χ^2^ = 2.55, *P* = .96, I^2^ = 0%), using fixed effect model analysis, RR = 0.71, 95%CI (0.51, 0.99), *P* = .04. It is suggested that the number of patients with abnormal liver function after treatment is lower than that in the control group (Fig. 7D).

The main adverse reactions of digestive system were nausea and vomiting, including 17 articles,^[13–16,18,20,21,24–26,30,32–34,37–39]^ homogeneity test (χ^2^ = 23.05, *P* = .11, I^2^ = 31%), fixed effect model analysis, RR = 0.69, 95%CI (0.61, 0.77), *P* < .00001, the difference was statistically significant. It is suggested that the number of nausea and vomiting in the treatment group is lower than that in the control group after treatment (Fig. 7E).

### 3.5. Publication bias

**Figure 8.**
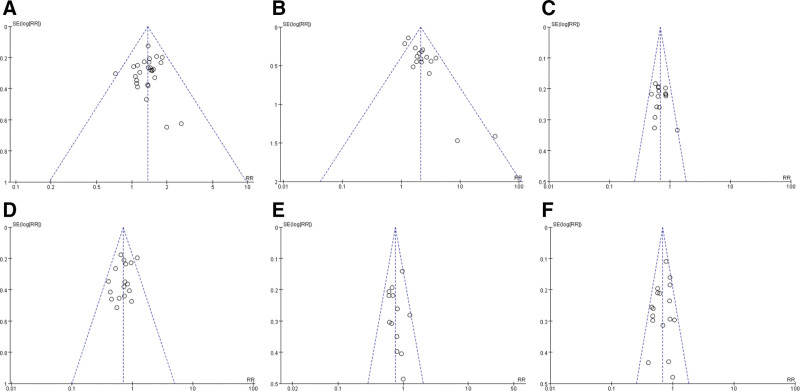
Funnel plots. (A: Funnel plot of Objective tumor response rate; B: Funnel plot of Quality of life score; C: Funnel plot of leukopenia; D: Funnel plot of thrombocytopenia; E: Funnel plot of hemoglobin decrease; F: Funnel plot of nausea and vomiting).

The publication bias of the study is tested by drawing the funnel chart, and the results show that the funnel chart is almost symmetrical, which shows that there is no obvious publication bias (Fig. 8).

## 4. Discussion

The early symptoms of NSCLC are not obvious, not easy to be detected, and are easy to be ignored to a large extent.^[41]^ When most lung cancer is found, it has developed to the middle and advanced stage, the therapeutic effect is not obvious, and the mortality rate is high.^[42]^ At present, chemotherapy is the main method for the treatment of advanced NSCLC, but the adverse reactions are substantial and inevitable. Therefore, adjuvant therapy is urgently needed to improve the quality of life and reduce adverse reactions. Traditional Chinese medicine has great advantages in adjuvant treatment of tumors. XAPI is the extract of Tongguanteng, a traditional Chinese medicine. According to the theory of traditional Chinese medicine, Tongguanteng has the effect of clearing away heat and detoxification, softening and dispersing knots. In clinic, XAPI, as an adjuvant drug for chemotherapy, is often used in the treatment of NSCLC. Modern pharmacological studies have shown that Tongguanteng and its preparations can inhibit cancer cell proliferation, regulate tumor cell angiogenesis, mediate apoptosis and promote differentiation, as well as synergistic and attenuating effects on other anticancer drugs. XAPI can promote the infiltration and function of CD8 (+) T cells,^[43]^ up-regulate tumor cell apoptosis-promoting genes and down-regulate suppressor genes to induce tumor cell apoptosis.^[44]^ XAPI has a synergistic effect on chemotherapy in the treatment of malignant tumors.^[45]^ However, there is still a lack of multicenter, large sample clinical studies to fully confirm the clinical efficacy and safety of XAPI.

A total of 28 clinical trials were included in this systematic review, all of which were RCTs. Meta analysis of random effects showed that the total effective rate, quality of life improvement rate and KPS score of Xiaoaiping injection combined with chemotherapy were better than those of chemotherapy alone. In terms of safety, the adverse reactions of digestive system, leukopenia, thrombocytopenia, hemoglobin and liver function after XAPI combined with chemotherapy were better than those of chemotherapy alone. In addition, in terms of immune function, XAPI combined with chemotherapy also has obvious advantages. This systematic review can show that the efficacy, safety and immune function improvement of XAPI combined with chemotherapy in the treatment of NSCLC is better than that of chemotherapy alone, and it can be considered in clinical application for patients with advanced NSCLC.

## 5. Conclusions

In conclusion, this study shows that compared with chemotherapy alone, XAPI combined with chemotherapy in the treatment of NSCLC can effectively improve the efficacy and safety of NSCLC by increasing ORR, improving KPS, immune function, liver function and reducing adverse reactions. Therefore, XAPI can be considered as a potential adjuvant therapy for patients with NSCLC. However, due to existing limitations, these conclusions require further large-scale RCTs to understand and confirm the long-term efficacy of XAPI.

## Author contributions

**Conceptualization:** Andong Li.

**Data curation:** Andong Li, Shilin Liu.

**Formal analysis:** Andong Li, Shilin Liu.

**Project administration:** Zhenyu Li, Jianan Xu.

**Software:** Hui Zhang, Minghao Lin.

**Supervision:** Lijiao Guo, Chengbo Yuan, Tan Wang.

**Writing – original draft:** Andong Li.

**Writing – review & editing:** Andong Li, Tan Wang.
